# Electrocardiographic Changes in Mitral Valve Prolapse Syndrome

**DOI:** 10.5681/jcvtr.2014.004

**Published:** 2014-03-21

**Authors:** Mohammad Mehdi Peighambari, Azin Alizadehasl, Ziae Totonchi

**Affiliations:** Rajaie Cardiovascular Medical & Research Center, Iran University of Medical Sciences, Tehran, Iran

**Keywords:** Mitral Valve Prolapse, Electrocardiogram, Early Repolarization

## Abstract

***Introduction:*** Mitral valve prolapse syndrome (MVP) is the most common valvular abnormalityin the young and is correlated with increased frequency of cardiac dysrhythmias and sudden death.The aim of this study was to compare frequency of “early repolarization” in electrocardiogram(ECG) between MVP patients and healthy adults.

***Methods:*** In this cross-sectional study, we compared ECG presentations of early repolarizationincluding notch in descending arm of QRS and J-point and/or ST segment changes in 100 patientswith MVP with 100 healthy individuals. MVP patients were referred to cardiology clinic withsymptoms of palpitation, chest pain or anxiety.

***Results:*** The mean age in patients with MVP was significantly less than healthy subjects (29.5 ±9.3 years versus 31.0 ± 6.9 years in control group, P= 0.1967). We detected early repolarizationas a prevalent sign in ECG of patients which was a notch in descending arm of QRS and/or STsegment or J-point elevation seen in 74% of patients ( 51% in inferior leads and 23% in I and aVLleads) , whilst the same findings were seen in 8 men (8%) in control group (P= 0.0001).

***Conclusion:*** Early repolarization in ECG presented as a notch in descending arm of QRS and/or ST segment or J-point elevation is more frequent in in young patients with MVP syndrome.

## Introduction

Mitral valve prolapse (MVP) is defined as superior displacement of the mitral valve leaflets more than 2 millimeters into the left atrium during systole and it is considered as the most common primary valvular abnormality in young populations.^1,2^ “Mitral valve prolapse syndrome” is often used to describe a constellation of MVP and associated symptoms like palpitation or any other physical abnormalities (autonomic dysfunction and funnel chest deformity).^1^ In some patients, mitral valve prolapse is silent or could be followed by palpitation, dizziness, chest pain, abnormal electrocardiogram findings and sometimes serious complications.^2-4^ Diagnosis can be confirmed by echocardiography and/or ventricular cineangiography, the latter permitting accurate recognition of the anatomy of the prolapsed leaflets. The complications of infective endocarditis, severe mitral insufficiency, and life-threatening ventricular arrhythmias represent the major problems of management.^3,4^ Many studies show some evidences of autonomic dysfunction^5^ and a source of arrhythmias^6^ including atrial^7^ and ventricular^8,9^ arrhythmias- specially in those with mitral regurgitation- or conduction disturbances^10^ in MVP patients. Other investigators reported P-wave dispersion and heart rate variability^11^ and ST-Segment Depression^12^ in patients presenting with MVP. Also some studies have reported arrhythmia induced sudden cardiac death in MVP patients.^13-16^ There is limited evidence about the signs of “early repolarization” (ER) in electrocardiogram (ECG) including notch in descending arm QRS and/or J-point or ST segment elevation in MVP syndrome, so in the present study we aimed to compare these signs between MVP patients and healthy individuals.

## Materials and methods

The study was performed after approving of the proposal in institutional ethic committee and obtaining informed written consent from all participants. In a cross-sectional study, we studied 100 patients with MVP syndrome diagnosis (according to echocardiography criteria) and 100 healthy subjects who referred to a university cardiology clinic. The echocardiography procedure was performed by two expert echocardiographists who agreed on the patient condition and they were examining patients blindly. Our echocardiographic criteria for MVP diagnosis was based on valve prolapse of 2 mm or more above the mitral annulus in the long-axis parasternal view and other views, especially when the leaﬂets coaptation occurred on the atrial side of the annular plane. Two patient-blinded cardiologists interpreted electrocardiographs taken from both groups after echocardiography procedure to detect signs of ER. ER was according to:

**1: J-point elevation** (presence and amplitude of ER). ER was defined as elevation of the J-point compared with baseline (the T-P segment). The J-point was defined as the earliest point of QRS offset (using a transparent ruler on paper ECG tracings, comparing all leads simultaneously). The J-point was measured in each lead separately. **2: ER-morphology-** In ER-present ECGs, the morphology was coded based on the visual appearance in reference to the attached example ECGs. **3: ST-morphology**- In ER-present ECGs, the morphology of the ST-segment was coded by comparing the amplitude 100 msec after the J-point separately in all ER-positive leads. The diagnosis was confirmed when all 2 interpreters had no conflict on diagnosis. Patients with coexisting cardiac disorders or a positive history for resuscitation after idiopathic ventricular fibrillation, or unexplained syncope or a familial sudden death at a young age were excluded from this study.

### Statistical methods

Numerical variables are presented as mean ± SD (standard deviation), and categorical variables are summarized by raw numbers and percentages. Continuous variables were compared using the independent samples *t*-test. Categorical variables were, on the other hand, compared using chi-square (with Yates correction) or Fisher’s exact test, as required. For the statistical analysis, the statistical software SPSS version 17.0 for windows (SPSS Inc., Chicago, IL, USA) was used. Statistical difference was considered significant if *P* value ≤ 0.05.

## Results

**Figure 1:**
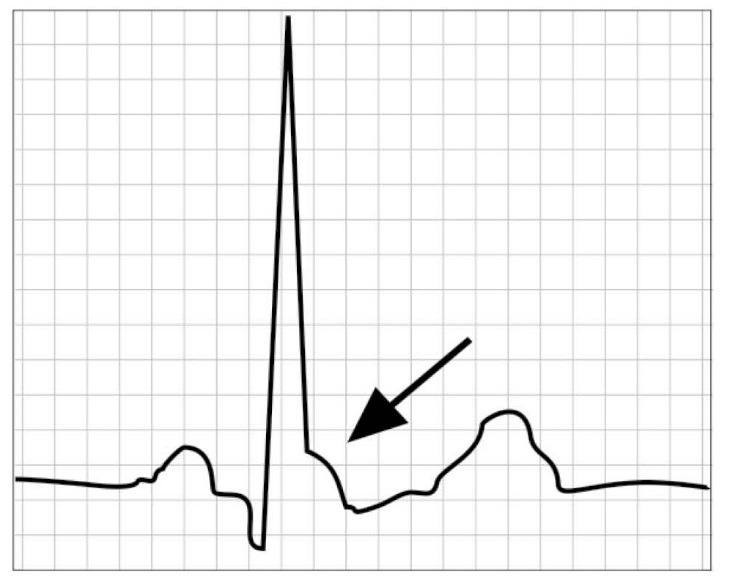
Notch in descending arm of QRS and J-point in lead II

Gender frequency in patients with MVP was 73 female versus 27 male, while in control group 65 patients were female and 35 were male, which showed to be statistically insignificant (*P*= 0.2845). Mean age of patients with MVP was 29.5 ± 9.3 years whereas it was 31.0 ± 6.9 years in control group (*P*= 0.1967). Clinical characteristics of MVP patients and healthy individuals (control group) including ECG parameters of ER were summarized in Table 1. In ECG examination, we observed that there was a notch in descending arm of QRS and/or ST elevation (early repolarization) that was seen in 74% of patients; (51% in inferior leads and 23% in I and aVL and 8% in all three mentioned leads) whereas this notch only was observed in 6 male patients in control group (*P*= 0.0001) (Figure 1).

**Table 1:** Clinical characteristics of mitral valve (MVP) patients and healthy individuals (control)

	**MVP Group** **n= 100**	**Control Group** **n= 100**	***P***
Age (year)	29.5 ± 9.3	31.0 ± 6.9	0.1967
Sex (M/F)	73/27	65/35	0.2845
Palpitation	64	4	0.0001
Chest pain	31	1	0.0001
Anxiety	75	22	0.0001
Ejection fraction (%)	59±7	55±9	0.0006
Early repolarization	74%	8%	0.0001
Inferior leads	51%	7	0.0001
I and aVL leads	23%	1	0.0001
In all inferior, I and aVL leads*	8%	1	0.0349

*These patients’ number was integrated into two above mentioned rows, so the total percentage of early repolarization in MVP group was 74.

Thin mitral valve leaflets was detected in major part of patient group (86%) which showed signs of early repolarization. However, the presence of early repolarization in remaining patients, it seems this sign to be independent factor than leflet thickness or presence of mitral regurgitation.

## Discussion

Electrocardiography has been proposed as a method to enhance the ability of the primary examination to detect underlying cardiac conditions.^17,18^ Early repolarization is more prevalent in young patients with MVP presenting with chest pain and anxiety and can be useful because of high prevalence of mitral valve prolapse and high incidence of presence of the sign on ECG. It seems that leaflet prolapse onto left atrium associated with papillary muscle traction is effective on the appearance of this finding. It seems impaired autonomic function is responsible for postural hemodynamic variations, cardiac arrhythmias and syncope in patients with MVP syndrome.^19,20^ It is not clearly known which mechanisms- either arrhythmogenic, neurohumoral or hemodynamic- leads to sudden death in this group of patients.^15^ Some pathophysiological studies demonstrated role of the vascular malformation and its effects on the cardiac chambers in sudden death in MVP.^15^ Length of anterior mitral valve leaflet and increased corrected QT dispersion are claimed to have significant correlation with ventricular arrhythmias in MVP.^21^

Also according to previous studies, early repolarization is the most common findings in patients with history of unexplained syncope, positive familial history of sudden death at young age or history of resuscitation after idiopathic ventricular fibrillation.^4,13-18^ In our study, we excluded all patients with such clinical history, yet we could detect signs of early repolarization in our patients. We can conclude that early repolarization is most common in MVP patients especially in inferior leads and could be a clue for diagnosis of MVP syndrome.

In order to assess specificity and sensitivity of this sign and the correlation between this finding with diverse symptoms, signs and outcomes as well as the reason for the variations of presence of this notch in inferior vs. I and aVL, we recommend an extended study in larger scales. Another goal of any future study could be to assess the correlations between the presence of early repolarization and ventricular and supraventricular arrhythmias.

## Ethical issues

The study was approved by the ethics committee of the University. Written informed consent was obtained from patients prior to the enrollment.

## Competing interests

Authors declare no conflict of interest in this study.
